# The Chronic Care Model and Diabetes Management in US Primary Care Settings: A Systematic Review

**DOI:** 10.5888/pcd10.120180

**Published:** 2013-02-21

**Authors:** Michael Stellefson, Krishna Dipnarine, Christine Stopka

**Affiliations:** Author Affiliations: Michael Stellefson, Christine Stopka, University of Florida, Gainesville, Florida.

## Abstract

**Introduction:**

The Chronic Care Model (CCM) uses a systematic approach to restructuring medical care to create partnerships between health systems and communities. The objective of this study was to describe how researchers have applied CCM in US primary care settings to provide care for people who have diabetes and to describe outcomes of CCM implementation.

**Methods:**

We conducted a literature review by using the Cochrane database of systematic reviews, CINAHL, and Health Source: Nursing/Academic Edition and the following search terms: “chronic care model” (and) “diabet*.” We included articles published between January 1999 and October 2011. We summarized details on CCM application and health outcomes for 16 studies.

**Results:**

The 16 studies included various study designs, including 9 randomized controlled trials, and settings, including academic-affiliated primary care practices and private practices. We found evidence that CCM approaches have been effective in managing diabetes in US primary care settings. Organizational leaders in health care systems initiated system-level reorganizations that improved the coordination of diabetes care. Disease registries and electronic medical records were used to establish patient-centered goals, monitor patient progress, and identify lapses in care. Primary care physicians (PCPs) were trained to deliver evidence-based care, and PCP office–based diabetes self-management education improved patient outcomes. Only 7 studies described strategies for addressing community resources and policies.

**Conclusion:**

CCM is being used for diabetes care in US primary care settings, and positive outcomes have been reported. Future research on integration of CCM into primary care settings for diabetes management should measure diabetes process indicators, such as self-efficacy for disease management and clinical decision making.

## Introduction

Diabetes is a major cause of heart disease and stroke among adults in the United States and is the leading cause of nontraumatic lower-extremity amputations, new cases of blindness, and kidney failure (1–3). In 2010, the Centers for Disease Control and Prevention reported that 25.6 million, or 11.3%, of US adults aged 20 or older had diagnosed or undiagnosed diabetes (1). Comprehensive models of care, such as the original Chronic Care Model (CCM) (4,5), advocate for evidence-based health care system changes that meet the needs of growing numbers of people who have chronic disease. CCM was developed (4,5) to provide patients with self-management skills and tracking systems. The model represents a well-rounded approach to restructuring medical care through partnerships between health systems and communities.

CCM comprises 6 components that are hypothesized to affect functional and clinical outcomes associated with disease management. The 6 components (4,5) are 1) health system — organization of health care (ie, providing leadership for securing resources and removing barriers to care), 2) self-management support (ie, facilitating skills-based learning and patient empowerment), 3) decision support (ie, providing guidance for implementing evidence-based care), 4) delivery system design (ie, coordinating care processes), 5) clinical information systems (ie, tracking progress through reporting outcomes to patients and providers), and 6) community resources and policies (ie, sustaining care by using community-based resources and public health policy).

The sum of these CCM component parts are purported to create more effective health care delivery systems that institute mechanisms for decision support, link health care systems to community resources and policies, deliver comprehensive self-management support services for patients, and operate and manage patient-centered clinical information systems. Despite evidence indicating widespread application of CCM to multiple illnesses, such as diabetes, congestive heart failure, and asthma (6), no summative reviews have investigated how CCM has been applied in diabetes care. The objective of this study was to determine how CCM has been applied in US primary care settings to provide care for people who have diabetes and also to describe outcomes of CCM implementation.

## Methods

### Data sources

This study identified English-language peer-reviewed research articles describing CCM-based interventions for managing type 1 and type 2 diabetes in US primary care settings (ie, hospital-network outpatient clinics, private practices, and community health centers). We collected articles from the Cochrane database of systematic reviews by using 2 distinct searches for “chronic care model” and “diabet*,” which were combined by using the word “and.” We also collected articles via EBSCOhost from the CINAHL database and the Health Source: Nursing/Academic Edition database by using the Boolean phrase search function for “chronic care model” (and) “diabet*.” These databases are all repositories for original health science research studies. Each database was separately searched. We conducted our analysis in October 2011.

### Study selection

Inclusion criteria specified that studies 1) be published after the formal inception of the original CCM (1999) (5); 2) use the original CCM (4,5) instead of the expanded CCM (7); and 3) describe CCM-based interventions to manage and treat diabetes in US primary care settings. We searched for articles published between January 1999 and October 2011. We excluded studies that took place outside of the United States, reported secondary data, or represented an editorial, commentary, or a literature review. We identified 155 studies (Figure) and reviewed them in 3 steps. First we reviewed the abstracts; 76 manuscripts met inclusion criteria, and 79 were excluded. We then reviewed the full articles; 43 articles were retained, and 33 were excluded. After additional review, we excluded 27 articles and retained 16 for data extraction.

**Figure:**
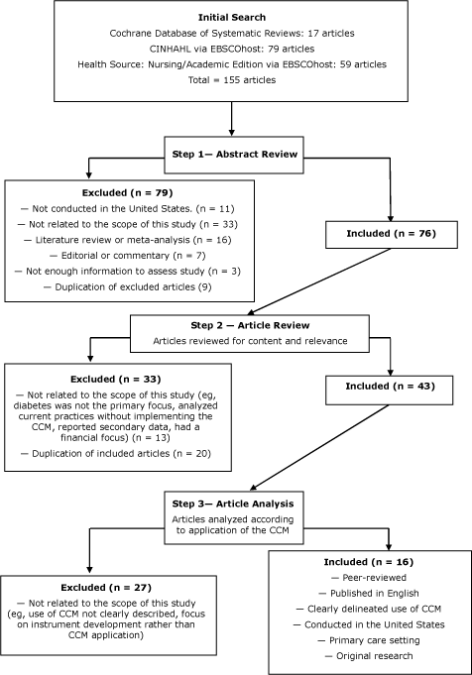
Manuscript selection for systematic review on the Chronic Care Model (CCM) and diabetes management in US primary care settings.

### Data extraction

As recommended by the Centre for Reviews and Dissemination systematic review guidelines (8), we created the following categories to systematically assess the 16 studies and gain an understanding of the methods used and the outcomes associated with CCM application: study design, sample size, setting, participant demographics, primary and secondary outcomes measured, data collection instruments used, statistical tests used, and major findings. We determined which of the 6 CCM components had been applied to each intervention and how the component(s) had been applied. We then qualitatively assessed the outcomes of each component that was applied in each study. The study selection process was conducted by 1 author (K.D.) and then repeated by the same author to ensure accurate selection; any discrepancies were analyzed and resolved on the basis of the inclusion and exclusion criteria. The same author (K.D.) extracted the data. Another author (M.S.) critiqued the data to identify any inconsistencies between data presented in the studies and the data extracted for the review, posed questions for further clarification on all extracted content, and then reviewed and synthesized the extracted data for accurate presentation within the context of the CCM.

## Results

The 16 studies (9–24) (Table 1) included 9 randomized controlled trials (9–17), 2 prospective cohort studies (18,19), 3 natural experiments (20–22), 1 qualitative study (23), and 1 cross-sectional study (24). Study settings included academic-affiliated primary care practices (10,12–14,21,23), private practices (11,16,17,20), community health centers (15,24), safety net clinics (18,19,22), and a hospital (9). Only 6 of 16 studies (11,12,16–18,20) implemented all 6 CCM components (Table 2). The studies focused primarily on people aged 50 to 70 years.

**Table 1:** Articles (N = 16) Included in a Systematic Review of the Chronic Care Model (CCM) Application for Diabetes Management

Study	Summary Data
**MacLean et al (** 9 **)**	
Study design (no. of participants)	Cluster randomized trial (n = 7,348).
Study setting	Patients from Vermont and New York primary care practices in the Vermont Diabetes Information System (VDIS).
Participant demographics	Mean age of patients, 62.9 y (range, 18–99 y).
Primary outcomes measured	VDIS effect on control of HbA1c levels.
Secondary outcomes measured	VDIS effect on patient satisfaction, medication use, lipids, renal function, blood pressure, functional status.
Instruments used	Medical Outcomes Trust SF-12 Health Survey, Audit of Diabetes-Dependent Quality of Life questionnaire, Self-Administered Comorbidity Questionnaire, Short Test of Functional Health Literacy in Adults, Primary Care Assessment Survey, Patient Health Questionnaire-9.
Statistical tests used	Generalized linear mixed model.
Major findings	A low-cost decision support and information system based on the CCM is feasible in primary care practices, especially practices that lack sophisticated electronic information systems.
**Siminerio et al (** 10 **)**	
Study design (no. of participants)	Multilevel, cluster-design, randomized controlled trial (n = 104).
Study setting	Rural primary care practice.
Participant demographics	Mean (SD) age, 65.4 (12.9) y; 99% white, 46% male.
Primary outcomes measured	Provider-perceived barriers to care, adherence to ADA standards of care, patient HbA1c, blood pressure, and cholesterol; patient knowledge and empowerment levels.
Secondary outcomes measured	None reported.
Instruments used	Barriers to Diabetes Care survey, Diabetes Attitude Scale, Diabetes Empowerment Scale, Diabetes Knowledge Test, and the Diabetes Self-Management Program of the University of Pittsburgh Medical Center Health System Initial Assessment.
Statistical tests used	Paired *t* test; McNemar test.
Major findings	Provider adherence to ADA guidelines improved significantly: lipid profile and urinalysis (*P* < .01); HbA1c measures (*P* < .001); dilated eye examination, foot examination, and monofilament (*P <* .001). Diabetes knowledge increased significantly from 67.3% to 78% (*P =* .003). Patients receiving DSME had significant improvements in HbA1c mean values (*P* = .007) and HDL cholesterol levels (*P* = .05). Patients who received DSME showed gains in all areas of empowerment: psychological, readiness to change, and goal setting. This study provides support for CDEs to receive financial reimbursement for services.
**Piatt et al (** 11 **)**	
Study design (no. of participants)	Multilevel, cluster-design, randomized controlled trial (n = 382).
Study setting	Private practices.
Participant demographics	CCM group mean (SD) age, 69.7 (10.7) y; 50% male; 13% nonwhite; 50% less than a high school diploma; 44% income <$20,000/year.
Primary outcomes measured	HbA1c, non-HDL cholesterol, blood pressure.
Secondary outcomes measured	Diabetes knowledge, empowerment, quality of well-being, frequency of blood glucose self-monitoring.
Instruments used	Diabetes Empowerment Scale, Modified Diabetes Care Profile, Diabetes Knowledge Test, World Health Organization (Ten) Quality of Well-Being Index.
Statistical tests used	Univariate analyses to determine differences between baseline and 12-month follow-up, paired *t* tests used for continuous data and McNemar test for categorical data. Between- and within-group analysis of variance used to examine differences among 3 study groups.
Major findings	Patients in the CCM group had significant increases in blood glucose self-monitoring at 12-month follow-up (*P* <.001). These outcomes were sustained at 3-year follow-up. HbA1c values declined significantly in the CCM group (7.6% to 7.0%, *P* = .008). A significantly greater (*P* = .04) proportion of participants in the usual-care group (54.2%), compared to the CCM (13.3%) and provider-education–only (38.9%) groups, had treatment intensification for glycemia.
**Siminerio et al (** 12 **)**	
Study design (no. of participants)	Multilevel, cluster-design, randomized controlled trial (n = 382).
Study setting	Suburban and urban primary care practices.
Participant demographics	Mean age, 57.2 y.
Primary outcomes measured	Patient HbA1c levels; number of ADA-recognized programs; proportion of patients who received DSME in primary care practices vs hospital-based programs; and reimbursement for CDE.
Secondary outcomes measured	None reported.
Instruments used	Laboratory results. The Medical Archival Retrieval System allowed for reimbursement and usability monitoring.
Statistical tests used	Student *t* test; Pearson χ^2^ test; Multilevel model for change.
Major findings	Number of ADA-recognized programs grew from 3 to 21 through decision support. A 2- to 3-fold greater proportion of patients reached when DSME was available at primary care practices compared to hospital-based programs. Having DSME programs at primary care practices resulted in improvements in HbA1c levels and better communication and use of resources among PCPs and CDEs. Patients reported comfort with location and ease of approaching CDEs.
**Smith et al (** 13 **)**	
Study design (no. of participants)	Physicians and their patients were randomized to the control or intervention group (clustered randomization). Physicians and patients were nonblinded, and outcome assessors and data analysts were blinded to allocation (n = 639).
Study setting	Academic-affiliated primary care practices.
Participant demographics	CCM intervention group that received virtual consultation: median duration of diabetes, 4 y (range, 0–43 y); median age, 62 y (range, 22–92 y); median BMI, 33 (range, 18–66); median HbA1c, 7.3 (range, 5.2–15.1).
Primary outcomes measured	Process of diabetes care, metabolic and vascular risk factor control with a 10-year estimated risk of cardiovascular disease; cost of care; participants’ functional health status.
Secondary outcomes measured	None reported.
Instruments used	Medical Outcomes Study Short Form 36. For process measurement: patients’ last visit to determine performance measures based on the ADA and National Committee on Quality Assurance Provider Recognition Program.
Statistical tests used	Generalized linear models.
Major findings	No significant differences in metabolic outcomes and coronary artery disease risk were found between control group and group receiving the virtual consultation.
**Stuckey et al (** 14 **)**	
Study design (no. of participants)	Randomized controlled trial (n = 549).
Study setting	Primary care clinics in Pennsylvania.
Participant demographics	Mean age, 58 y; 57% female; 39% Hispanic.
Primary outcomes measured	Percentage of patients achieving goals for HbA1c, blood pressure, and LDL cholesterol.
Secondary outcomes measured	Number of patients with depression; rates of eye and foot examinations; nephropathy assessment; cost-effectiveness; psychological and behavioral outcomes.
Instruments used	Audit of Diabetes Dependent Quality of Life survey, Problem Areas in Diabetes scale, Diabetes Treatment Satisfaction Questionnaire, Summary of Diabetes Care Activities, and the Provider Satisfaction Inventory.
Statistical tests used	Logistic regression for binary outcome measures (eg, success in meeting HbA1c, blood pressure, and LDL cholesterol goals); generalized estimating equations for longitudinal data; repeated measures analysis of variance for continuous outcomes (eg, HbA1c, systolic blood pressure, lipids).
Major findings	Study in progress, so other than baseline data, outcomes have not been reported. Baseline survey scores of the patient population showed a high level of depression and a slightly positive effect of diabetes on self-confidence and that diabetes had most negative effect on enjoyment of vacations and on enjoyment of food and drinks.
**Schillinger et al (** 15 **)**	
Study design (no. of participants)	Practical clinical trial with 3 arms: interactive weekly automated telephone self-management support with nurse follow-up (ATSM), group medical visits with physician and health educator facilitation (GMV), and usual care; random assignment to groups (n = 339).
Study setting	County-run clinics in the Community Health Network of San Francisco.
Participant demographics	Mean (SD) age, 56.1 (12.0) y.
Primary outcomes measured	1-year change in self-management behavior.
Secondary outcomes measured	Degree of structure of care alignment with CCM; process of care alignment with CCM; patient weekly self-care, quality of life, days spent in bed because of health problems; effect of diabetes on activities of daily living.
Instruments used	Patient Assessment of Chronic Illness Care, short-form Test of Functional Health Literacy in Adults, Interpersonal Processes of Care for Diverse Populations, Summary of Diabetes Self-Care Activities Measure, Diabetes Quality Improvement Program diabetes self-efficacy measure, Short Form-12 instrument for quality of life.
Statistical tests used	Paired *t* test; McNemar test.
Major findings	ATSM seems to be a more effective communication method for self-management support than monthly GMV for improving behavior and quality of life for patients with poorly controlled diabetes. ATSM group had significant decreases in days restricted to bed compared with usual care group (−1.7 days per month, rate ratio 0.5 [95% CI, 0.3–1.01]). ATSM group was less likely than GMV and usual care groups to report that diabetes prevented them from carrying out daily activities. No significant changes in HbA1c were found in ATSM, GMV, and usual-care groups.
**Piatt et al (** 16 **)**	
Study design (no. of participants)	Multilevel, nonblinded, cluster design, randomized controlled trial (n = 119).
Study setting	Private practices divided into 3 groups: CCM, provider education only, and usual care.
Participant demographics	CCM group mean (SD) age, 69.0 (12.3) y; 53.3% male, 20% nonwhite.
Primary outcomes measured	HbA1c, non-HDL cholesterol, and blood pressure levels at 3-year follow-up.
Secondary outcomes measured	Sustained outcomes in quality of well-being, self-monitoring of blood glucose.
Instruments used	Modified Diabetes Care Profile; World Health Organization (Ten) Quality of Well-Being Index.
Statistical tests used	Paired *t* test; McNemar test.
Major findings	HbA1c improvements observed at 1-year follow-up were sustained in 8 of 12 participants in CCM group at 3-year follow-up, whereas the provider-education–only group and usual-care group remained constant from baseline. Mean non-HDL cholesterol values and systolic and diastolic blood pressure improved in all groups, although the only statistically significant improvement was in diastolic blood pressure in the CCM group (*P=*.04).
**Piatt et al (** 17 **)**	
Study design (no. of participants)	Multilevel, cluster-design, randomized controlled trial (n = 119).
Study setting	General, family, and internal medicine practices (n = 24) in Pittsburgh, Pennsylvania.
Participant demographics	Means for CCM, provider-education–only, and usual-care groups combined: mean (SD) age, 67.6 (9.4) y; 50.4% male; 8.6% nonwhite.
Primary outcomes measured	Provider-perceived patient barriers to care; adherence to ADA standards of care; patient HbA1c, blood pressure, non-HDL cholesterol levels; height and weight; knowledge and empowerment levels; diabetic, lipid and blood pressure treatment intensification.
Secondary outcomes measured	None reported.
Instruments used	Barriers to Diabetes Care Instrument, Diabetes Empowerment Scale, and the World Health Organization (Ten) Quality of Well-Being Index.
Statistical tests used	Forward linear regression, general linear regression.
Major findings	The CCM group had the largest decrease in HbA1c values (−0.6%, *P* = .008) compared with no significant reduction in the provider-education–only and usual-care groups, with no significant change in treatment intensification. Having higher baseline HbA1c values, older age, and being in the CCM group were each associated with improved glycemic control.
**Stroebel et al (** 18 **)**	
Study design (no. of participants)	Prospective cohort study (n = 149).
Study setting	Free medical clinic for uninsured patients.
Participant demographics	76 female patients; mean age, 51.9 y; 47.7% Hispanic.
Primary outcomes measured	Clinically significant improvement for patients in at least 1 chronic disease (ie, 1-stage reduction in blood pressure for hypertensive patients, decrease of at least 1% of HbA1c for patients with diabetes, reduction of risk group in LDL cholesterol for patients with hyperlipidemia).
Secondary outcomes measured	Change in mean arterial pressure, change in HbA1c, change in LDL cholesterol.
Instruments used	Laboratory results, clinical measures.
Statistical tests used	Paired *t* test.
Major findings	64% of patients with hypertension improved by at least 1 stage; 53% had a 1% reduction in HbA1c levels; 58% of patients with high LDL cholesterol improved by 1 risk group; mean arterial pressure, mean HbA1c, and mean LDL cholesterol showed significant improvements (*P <* .001); CCM was found to be a successful template for delivering chronic care to uninsured patients in a free medical clinic.
**Khan et al (** 19 **)**	
Study design (no. of participants)	Prospective single cohort study (n = 1,098).
Study setting	Walk-in urgent care clinic for uninsured patients.
Participant demographics	Mean (SD) age, 51 (12) y; 59% male, 42% African American.
Primary outcomes measured	Health beliefs, self-reported dietary habits, weight, HbA1c, systolic and diastolic blood pressure, LDL cholesterol, patient satisfaction with clinic.
Secondary outcomes measured	None reported.
Instruments used	Laboratory results, self-reported dietary habits and health beliefs, patient satisfaction with clinic rated on scale of 1 to 10.
Statistical tests used	McNemar test for dichotomous data, Wilcoxon signed rank test for ordinal data, and paired *t* test for continuous data.
Major findings	Mean change in lowering HbA1c levels was significant (*P <*. 001); systolic blood pressure decreased on average by 9 mm Hg; diastolic blood pressure decreased on average by 5 mm Hg; LDL cholesterol decreased on average 16 mg/dL; 80% of patients rated satisfaction with clinic as 8 or higher.
**Benedetti et al (** 20 **)**	
Study design (no. of participants)	Natural experiment with comparison group; 11 participating providers had 698 patients; 19 nonparticipating providers had 1,300 patients.
Study setting	Private-sector, fee-for-practice, multispecialty group practices.
Participant demographics	Not described.
Primary outcomes measured	HbA1c, blood pressure, LDL cholesterol, urine protein, rates of eye and foot examinations, acetylsalicylic acid intake for patients age >40 y, and provider satisfaction.
Secondary outcomes measured	None reported.
Instruments used	Laboratory results; provider satisfaction survey.
Statistical tests used	2-Tailed *t* test; *t* test for equal or unequal variances, depending on an equality of variances test. *F* tests used to determine whether length of time in the collaborative model affected outcomes.
Major findings	Favorable adherence to eye examinations and blood pressure control associated with increased time (in years) of provider participation using CCM (*P* < .05). Similar trends found in patients taking acetylsalicylic acid, having foot examinations, setting goals for self-management, having annual HbA1c test, having an HbA1c < 8.0, and having an annual urine protein test. 78% of providers expressed satisfaction with their collaborative work after using CCM; only 28% expressed satisfaction before implementing CCM.
**Coca and Francis (** 21 **)**	
Study design (no. of participants)	8-month pilot test, natural experiment (n = 48).
Study setting	University-based care delivery system.
Participant demographics	Participants had an established diagnosis of type 2 diabetes (age and sex were not reported).
Primary outcomes measured	Blood pressure; HbA1c levels; documentation and follow-up of goal setting; eye and foot examinations; medical residents receiving/reviewing/discussing registry reports; medical residents learning and demonstrating self-management support strategies.
Secondary outcomes measured	Vaccinations, medications (statin use, angiotensin-converting enzyme inhibitor/angiotensin receptor blocker, aspirin), microalbuminuria.
Instruments used	Laboratory results.
Statistical tests used	None reported. Percentage improvement was calculated.
Major findings	Participants showed improvement in performance measures, such as initiating goal setting, receiving eye and foot examinations, seeking vaccinations, attaining blood pressure goals, and adhering to medication instructions, but they showed nonsignificant improvement in HbA1c.
**Caruso et al (** 22 **)**	
Study design (no. of participants)	Natural experiment (n = 283).
Study setting	Safety-net hospital.
Participant demographics	Mean (SD) age, 76.0 (8.6) y; 64% black; 9% Hispanic; 15% white; 10% other race/ethnicity; 72% had Medicare; 3% had Medicaid; 6% had other insurance or payment methods; 19% state-subsidized care.
Primary outcomes measured	HbA1c, foot examinations, lipid panel, blood pressure, number of patients who had cardiovascular disease, diabetes, or both.
Secondary outcomes measured	None reported.
Instruments used	Laboratory results.
Statistical tests used	Paired *t* tests for continuous variables; contingency tables and McNemar χ^2^ tests for categorical variables across intervention periods; multivariate analysis using generalized estimating equations to evaluate change over time.
Major findings	The low-cost and time-efficient interventions used in this study (ie, developing a protocol for foot examinations, training patients and medical assistants in foot examination, and tracking patients for follow-up appointments) improved clinical outcomes (blood glucose, lipid, blood pressure, and foot examinations) of patients who had both diabetes and cardiovascular disease.
**Lyles et al (** 23 **)**	
Study design (no. of participants)	Qualitative thematic analysis of semistructured interviews (n = 14).
Study setting	University of Washington general internal medicine clinic, Seattle, Washington.
Participant demographics	Age range, 18–75 y (mean not provided); all participants had HbA1c greater than 7%.
Primary outcomes measured	Process measures: glucose readings and uploads, patient–provider e-mails.
Secondary outcomes measured	Participant satisfaction.
Instruments used	Qualitative interview guide.
Statistical tests used	Phenomenology to analyze participant’s narratives; thematic coding; Atlas.ti version 5.2 used to analyze relationship between concepts and analyze codes across transcripts.
Major findings	Study produced mixed results. Patients felt more aware of and engaged in their own care through monitoring their glucose, sharing their glucose readings with the nurse case manager, and communicating with the nurse case manager via the secure e-mail system; uploading glucose readings and receiving feedback was easy. However, half of the patients found the use of smartphones to be frustrating (unfamiliar technology). Using the Nintendo Wii to access electronic medical records was not useful (unfamiliar technology).
**Liebman et al (** 24 **)**	
Study design (no. of participants)	Cross-sectional (n = 275).
Study setting	Community health center.
Participant demographics	Of 6 years of data in the diabetes registry, 5% aged <30 y, 39.7% aged 30–64 y, 15.5% ≥65 y.
Primary outcomes measured	Glycemic control; patient participation in activities.
Secondary outcomes measured	None reported.
Instruments used	Laboratory results, participation data from registry.
Statistical tests used	Statistical analysis was not described.
Major findings	HbA1c levels were consistent for 4 years before implementation of self-management activities. Participants showed a decrease in HbAlc levels (mean HbA1c decreased from 8.6 to 8.0) after an average of 20.6 months of participation in self-management activities. At the end of the study, nearly half of the center’s patients with diabetes reached the target goal of an HbA1c less than 7.0.

Abbreviations: SD, standard deviation; ADA, American Diabetes Association; HbA1c, hemoglobin A1c; DSME, diabetes self-management education; CDE, certified diabetes educator; HDL, high-density lipoprotein; PCPs, primary care providers; BMI, body mass index; LDL, low-density lipoprotein; CI, confidence interval.

**Table 2:** Application of the Chronic Care Model (CCM) Components^a^ for Diabetes Management in the 16 Studies Included in the Systematic Review

Study/Component	Application
**MacLean et al (** 9 **)**	
Decision support	Developed the Vermont Diabetes Information System to collect clinical information and provide flow sheets, reminders, and alerts to physicians and their patients with diabetes. The system also generates population reports so that physicians can view the progress of their patients with diabetes.
	CCM is used as the framework; laboratories provide daily data feeds; algorithms provide automatic test interpretation; fax and mail are used for providers not easily reached by electronic networks; reports are formatted for accessibility and usability by patients and providers.
**Siminerio et al (** 10 **)**	
Self-management support	Participants and their family members met with team members for five 2-hour group sessions biweekly. Each group consisted of 5 to 10 participants who learned goal-setting strategies based on the empowerment approach, problem-solving skills, and behavioral change strategies.
	Patients led the discussion according to individual needs, and the CDE facilitated the discussion to include ADA’s 10 content areas.
Decision support	PCPs were trained by CDEs on ADA standards of care and implementation of guidelines.
	Problem-based learning sessions were used to demonstrate implementation of guidelines into a plan of care.
	PCPs completed routine examination and assessed complications during each visit.
	Process delivery (HbA1c, lipid panel, blood pressure, urinalysis, dilated eye referral, foot examination, and use of monofilament) were to be recorded by PCPs.
Delivery system design	“Diabetes days” were organized: on these days, CDEs were in PCP offices for routine office visits and DSME.
**Piatt et al (** 11 **), Piatt et al (** 16 **), Piatt et al (** 17 **)**	
Health system — organization of health care	Principal investigator met with PCPs to determine needs.
	Funding was obtained from local hospital foundation and parent hospital system.
Self-management support	Offered 6 weekly CDE-facilitated DSME sessions based on the University of Michigan DSME curriculum.
	Monthly support groups focused on foot care, healthful cooking and recipe modification, alternative treatments, and problem-solving skills.
	Used ADA diabetes education content areas.
	Used empowerment approach during patient visits.
Decision support	Problem-based learning sessions were held for PCPs, led by an endocrinologist using diabetes management questions.
	PCPs received training on ADA standards of care for people with diabetes.
	Flow sheets, which incorporated ADA guidelines to track patient testing and results, were provided.
Delivery system design	“Diabetes days” scheduled; on these days, a CDE was present in PCP offices.
	PCPs were encouraged to refer patients to CDEs whenever possible.
Clinical information systems	Most PCPs did not have computers or electronic medical records.
	Baseline chart audit was conducted to establish benchmark for adherence to ADA standards of care and enhance provider feedback.
Community resources and policies	Collaborations were formed between the University of Pittsburgh, community leaders, physicians, community hospital foundation, and Lions clubs.
**Siminerio et al (** 12 **)**	
Health system — organization of health care	University of Pittsburgh Medical Center provided educators with access to funding, information systems, PCPs, and hospital administration.
Self-management support	Provided tracking forms and education materials.
	Met ADA recognition qualifications for diabetes educator support in PCP offices.
	Structured DSME was based on ADA education content areas.
	DSME initially delivered on an individual basis; group visits were facilitated later in the study as office space became available.
Decision support	The University of Pittsburgh Medical Center supported the implementation of ADA standards of care, covered fees for the application for ADA recognition, supported the development of a central coordination center for educators, supported seminars for training and certification, supported the development of a central advisory committee, which included representatives from hospital sites, the community, and physician practices.
Delivery system design	CDE worked with staff to schedule DSME; CDE served as a clinical resource; PCPs hosted “diabetes days”; PCPs made direct referrals to CDEs.
Clinical information systems	Medical Archival Retrieval System was used to track reimbursement, DSME service rates, and HbA1c levels.
Community resources and policies	The University of Pittsburgh Medical Center facilitated communication and sharing of resources between diabetes educators and administrators in community hospitals and primary care practices.
**Smith et al (** 13 **)**	
Decision support	An electronic library of messages was developed according to best available research on the use of aspirin, angiotensin-converting-enzyme inhibitors, and angiotensin receptor blockers and management methods for glycemic control, diet and exercise, dyslipidemia, hypertension, chronic heart failure, and nicotine dependence.
	This information was used to create single-line, positively framed messages (information was presented as gains, not losses), which were shown to elicit a better response from physicians. These messages had links to relevant references for more in-depth information.
	Endocrinologists provided the telemedicine intervention, delivering these tailored messages to the primary care team for review 48 hours before the patient’s next scheduled visit.
	The PCP and patient reviewed the message and decided how to proceed.
	The PCP then answered yes or no if the message was helpful and if it was used in developing patient plans.
**Stuckey et al (** 14 **)**	
Health system — organization of health care	Nurse case managers were integrated into a primary care setting to work with study participants, PCPs, endocrinologist, diabetes educator, and dietitian.
Self-management support	Motivational interviewing was used with patients, and self-management education was provided through a CDE.
Decision support	Nurse trained on ADA clinical care guidelines.
Delivery system design	Case management, evidence-based care, cultural competency, improved provider interactions.
Clinical information systems	Penn State Institute for Diabetes and Obesity patient registry system was used to identify patients with uncontrolled diabetes (HbA1c >8.5), hypertension (blood pressure >140/90 mm Hg), or hyperlipidemia (low-density lipoprotein cholesterol >130 mg/dL). Nurses also entered patient information into the registry, and single-sheet patient reports could be generated from the registry to show self-care goals, patient’s trends (eg, blood pressure, HbA1c, lipids, eye examination, aspirin use, foot examination), and alerts for issues to address during the patient’s visit (eg, missed examination, abnormal laboratory results).
**Schillinger et al (** 15 **)**	
Self-management support	Patients had either interactive weekly automated telephone self-management support with nurse follow-up or monthly group medical visits with physician and health educator facilitation.
**Stroebel et al (** 18 **)**	
Health system — organization of health care	Project was fully supported by the governing board of the Salvation Army Free Clinic.
Self-management support	Collaborative goal setting addressed self-monitoring and lifestyle modification by using a self-management wheel to display components.
	Nurses followed up with telephone calls to monitor progress toward goals.
Decision support	Used Institute for Clinical Systems Improvement Clinical Guidelines for Hypertension, Diabetes, and Hyperlipidemia.
	Core physicians were advocates of guideline-based management.
	Specialty expertise from a volunteer endocrinologist was consistently available by telephone or e-mail.
	CDE met with patients who had diabetes.
Delivery system design	Nurses interacted most with the patients, using evidence-based algorithms from the Institute for Clinical System Integration to provide patient care and manage medications.
	Telephone and e-mail communication facilitated interaction between nurses, volunteer physicians and specialists (eg, endocrinologists). The volunteer physicians and specialists were available for consultation to manage challenging cases and questions (eg, difficult medication issues, questions directed to the physicians).
	Medications were available at no cost to patients according to clinic policy and practices.
Clinical information systems	Secure, password-protected patient registry was created on Microsoft Excel and managed by a registered nurse.
Community resources and policies	Salvation Army Free Clinic was a product of community collaboration and the volunteer efforts of professionals and community laypersons.
**Khan et al (** 19 **)**	
Health system — organization of health care	Clinic space was modified to provide services.
	Staff were reorganized and retrained to provide chronic care.
Self-management support	Educational materials were developed for patients with diabetes.
	30-Minute interactive group sessions focused on dietary choices, exercise, weight loss, and self-monitoring.
	Medications were reviewed in group setting; discussion focused on adherence.
	Computer-based educational modules focused on diabetes self-management topics.
	“Lifestyle school” session included a model grocery store so participants could practice reading food labels, learn and apply skills to choose more healthful options during grocery shopping and when considering fast food options.
	“Diabetes Passport” served as patient’s personal record of blood pressure, HbA1c levels, weight, and cholesterol, along with their goals and plans.
	Nurses worked with patients to complete a computer program to calculate 10-year risks for heart, vascular, renal, and eye disease on the basis of individual patient factors. A discussion of possible behavior changes followed, concluding with agreed-upon goals. This information was reviewed during the patient’s scheduled follow-up visit to assess retention of the information learned in the previous visit and progression toward the set goals.
Decision support	Used evidence-based treatment protocols.
	Staff received training for new roles in chronic care.
Delivery system design	Group educational sessions consisting of 5 to 25 patients motivated patients to engage in positive behavior change and to apply problem-solving skills.
	Computer-based educational sessions were conducted individually or in small groups; patients were given unlimited walk-in access so they could actively engage in learning about and controlling their conditions.
	Clinic nurse assisted patients with computer program to assess 10-year risks and focus on behavior change and goal-setting.
Clinical information systems	Comprehensive electronic database consisted of data on patient interviews, examination and laboratory results, habits, attitudes, goals, medication use, and follow-up visit plans.
**Benedetti et al (** 20 **)**	
Health system — organization of health care	Job descriptions of the medical director and quality improvement coordinator were altered to include improvement in the care of patients with chronic illnesses.
	Rockwood Clinic Foundation mission was refocused toward efforts to support and promote research in new systems of health care delivery.
Self-management support	Created patient self-management toolkit.
	Implemented patient goal-setting strategies and group visits.
Decision support	Improved CDE referral system; gave clinical teams monthly reports to track patient performance; clinical teams meet quarterly to review results and receive clinical information updates.
Delivery system design	Hosted planned visits every 3 months for PCPs to focus primarily on patients with diabetes; organized group visits with 10 to 12 patients and 3 care team members per session; revised team roles to include greater focus on proactive involvement in patients’ care.
Clinical information systems	Created patient registry to track clinical measures and generate patient performance reports for patients and providers.
Community resources and policies	Developed collaborations with pharmaceutical companies and health plans; hosted community health fair focused on diabetes; provided community PCPs with training sessions on using the CCM for diabetes.
**Coca and Francis (** 21 **)**	
Self-management support	Used a self-management goal sheet to set patient goals, estimate patient’s confidence in achieving his or her goal from “not confident” to “very confident,” and check on goal adherence with a 2-week follow-up call by a resident.
	Used goal-setting and motivational interviewing strategies.
Decision support	“Planned Visit Worksheet” was used to ensure evidence-based diabetes care aspects were addressed during visits.
	Follow-up visits were scheduled after each planned visit according to the patient’s degree of diabetes control.
Delivery system design	Each resident practiced a planned visit with a patient.
	Registry was used to identify patients who had not been seen in 6 months or had HbA1c levels >8%.
Clinical information systems	Patient registry was a major advancement for identifying patients, generating individual and private reports, and developing Plan-Do-Study-Act (PDSA) cycles for diabetes care.
**Caruso et al (** 22 **)**	
Self-management support	Provided pamphlets on cardiovascular disease and diabetes.
	Tips were displayed on bulletin boards.
	Patients received folders that included information about their disease, disease-specific self-management skills, and doctor-patient communication skills.
Decision support	Training sessions were held for all providers and staff.
	CDE trained medical assistant to conduct foot examinations.
Delivery system design	CDE provided patient and provider education and service onsite.
Clinical information systems	Used electronic medical records and flow sheets, which were valuable for contacting patients who have not been seen in a while, and in following the performance and progress of patients (eg, results for HbA1c, low-density lipoprotein cholesterol, blood pressure, foot examinations).
**Lyles et al (** 23 **)**	
Self-management support	Patients used a home computer or their Nintendo Wii game system to review their electronic medical record through the My Health Record interface.
	Blood glucose readings were remotely uploaded to providers for interactive feedback through a wireless Bluetooth device connecting a glucometer and a smartphone.
	Interactive feedback using the Web-based My Diabetes Daily Diary self-management tool focused on nutrition, medications, and exercise.
	Patients communicated with a nurse case manager on their diabetes care via a secure e-mail connection.
	Provided a general diabetes educational website that included links to information endorsed by the University of Washington Diabetes Care Center medical director.
Decision support	Interactive electronic medical record was shared by patient and provider. Accessible to patients on a personal computer or a Nintendo Wii, it provided clinical reminders and patient performance summaries.
Delivery system design	Used nurse case managers in the diabetes care delivery process; provided proactive follow-up based on patient needs, including the development of action plans to meet patient diabetes care goals; used information exchanged via secure e-mail communication between the nurse case manager and the patient to enhance patient care during office visits; integrated blood glucose trends and lifestyle information into ongoing patient care.
Clinical information systems	Ongoing tracking and documentation of patients’ needs and care process.
	Provided access to electronic shared medical record for patients and providers; included secured e-mail for interactive feedback with case managers.
**Liebman et al (** 24 **)**	
Self-management support	Offered weekly breakfast club focused on nutrition and cooking skills and healthful modifications for traditional Puerto Rican recipes.
	Offered weekly afternoon snack club to teach participants about healthful snack preparation and reinforce problem-solving and self-management skills.
	Offered weekly diabetes education classes for 11 weeks using the curriculum developed by the Midwest Latino Health Research Center and including a supermarket tour.
	Offered chronic disease self-management classes to teach patients behavioral goal setting and strategies to overcome barriers and promote peer support.
	CDE provided individual diabetes counseling, including nutritional counseling.
	Offered daily onsite exercise classes.
	Provided bilingual/bicultural community health workers’ services, including home visits, accompanying patients on medical visits, and telephone and in-person counseling and support.
Decision support	Trained clinicians to treat patients to target blood glucose control and cardiovascular risk factors.
	Developed protocol to provide clinicians with key clinical information for each patient visit.
Delivery system design	Team approach to care delivery used clinicians, nurses, and medical assistants.
Clinical information systems	Electronic registry of patients with diabetes tracked care and outcomes.
Community resources and policies	Patients were linked to available community resources.

Abbreviations: CDE, certified diabetes educator; ADA, American Diabetes Association; PCP, primary care physician; DSME, diabetes self-management education.

a The 6 components of the CCM are 1) health system — organization of health care, 2) self-management support, 3) decision support, 4) delivery system design, 5) clinical information systems, and 6) community resources and policies.

### Health system — organization of health care

Support from health care leaders stimulated organizational changes (9–12,14,16–22,24). Engaging the governing boards of health care systems resulted in support for institutionalizing the CCM approach (18,22), which was associated with HbA1c reductions of at least 1% during 12 months (18,22) and improved foot care (22). Two studies (19,20) revised the health care system to redefine health care team roles (eg, nurses, instead of PCPs, became responsible for conducting foot examinations). These changes improved the quality of diabetes care and rates of eye examinations, and were associated with improved HbA1c levels, blood pressure, cholesterol, and weight (19,20). Health system reorganization also helped to establish diabetes self-management training programs (12,16,17) that identified and intervened with patients at risk for developing complications (17) and improved clinical and behavioral outcomes (12,16).

### Self-management support

We found that diabetes self-management education (DSME) generally improved psychosocial and clinical outcomes in patients with diabetes. Twelve of 16 studies administered individual DSME sessions (10–12,14–21,24), and 9 studies (10–12,15–17,19,20,24) administered group sessions using both group- and individual-level approaches. Facilitators, such as Certified Diabetes Educators (CDEs) or nurses, provided instruction on various topics, such as medication compliance, goal setting, foot care, and interpretation of laboratory results (10–12,14–17,20,24). Follow-up telephone calls allowed clinicians to monitor patient progress toward meeting diabetes-management goals that were set during individual office visits (10,15,18,21). For example, Schillinger et al (15) found that weekly automated (prerecorded) tailored telephone calls from nurses were associated with improvements in interpersonal processes of care, physical activity and function, and slightly better metabolic outcomes (eg, HbA1c, blood pressure, cholesterol). Lyles et al (23) found that the use of a secure e-mail connection and a smartphone to upload glucose readings via a wireless Bluetooth device allowed some participants to feel better connected with their nurse case manager. However, some participants found this communication system to be unstructured and preferred regular interaction (eg, face-to-face) with their nurse case manager; some participants found the smartphones to be frustrating because of technical difficulties associated with these unfamiliar technologies (23). Other studies reported that computer-based interactive diabetes self-management training modules and toolkits were supplemented by a “diabetes passport” (19) or “diabetes care record” (20) that listed goals, action plans, and laboratory results so that patients and providers could monitor performance and progress in diabetes care.

### Decision support

Specialized decision support services for diabetes care were provided to PCPs (eg, endocrinologists) and nurse practitioners via telephone and e-mail (18), problem-based learning meetings (11,12,14,16,17), and telemedicine technology (13). Individual patient reports were also provided to health care teams for reviewing clinical trends (eg, HbA1c, blood pressure, lipids) and initiating clinical responses to laboratory results (eg, medication adjustments) (9,10,20,23). Training PCPs on evidence-based guidelines and methods for implementing CCM resulted in improved PCP adherence to clinical guidelines, including the American Diabetes Association (ADA) Standards of Care (10–12,14,16,17) and Institute for Clinical Systems Improvement (ICSI) Clinical Guidelines for Hypertension, Diabetes, and Hyperlipidemia (18). In several studies (10–12,14,16–18), this training was associated with improved diabetes knowledge among patients and improved levels of HbA1c and high-density lipoprotein (HDL) cholesterol.

### Delivery system design

Implementation of ADA standards of care (10–12,14,16,17) and ICSI clinical guidelines (18) resulted in innovative diabetes care delivery in PCP offices. For example, ADA standards require that people with diabetes receive DSME to “optimize metabolic control, prevent and manage complications, and maximize quality of life in a cost-effective manner” (25). To address barriers to care, such as poor diabetes knowledge, low awareness of educational service accessibility, and lack of psychosocial support (10,26), PCPs streamlined DSME services by offering “diabetes days” and planned visits exclusively for people with diabetes (10–12,14–21,24). Instituting these programs in PCP offices allowed for better communication between CDEs, PCPs, and patients, which contributed to lower HbA1c levels (10–12,18,20,24); better adherence to medication and adjustment processes; and stronger support networks located in more personalized settings (10,11,15–17,19,20,24). One study (12) even noted that providing DSME programs in PCP offices instead of hospital settings resulted in a 2- to 3-fold increase in the number of patients reached with diabetes education.

### Clinical information systems

Collaborative clinical information systems using disease registries and electronic medical records enabled multiple health care providers (eg, PCPs, nurse practitioners, nurses, CDEs, physician assistants, medical assistants) to review detailed reports on laboratory and examination results and identify lapses in diabetes care (eg, missed visits, laboratory appointments, and examinations). These systems helped patients and providers set self-management goals and review progress reports to determine whether patients met their predetermined goals (9,11,12,14,16–18,20,21,23,24). Improved tracking (ie, using electronic patient registries or electronic medical records) of individual health outcomes (eg, HbA1c trends) provided an expedient way to manage patient information (9,12–15,18–23) and also improved provider responses (eg, medication adjustment) to clinical data (9,10,13–15,18–23). For example, the Medical Archival Retrieval System (MARS) stored data and generated robust reports for providers on laboratory results, visits, medications, health insurance, comorbid conditions, medical procedures, and billing charges (12). MARS also served as a tool for administrators to gauge fiscal outcomes associated with placing CDEs in primary care sites to deliver DSME (12).

### Community resources and policies

Seven studies (11,12,16–18,20,24) specified strategies for using community resources and forming public policy. Collaborations between community leaders and physicians (11,16,17) and between pharmaceutical companies and health plans (20) led to support for PCP training sessions on how to use CCM for diabetes management. Hospital and PCP collaborations within the community, such as partnerships between the University of Pittsburgh Medical Center and western Pennsylvania community hospitals and PCP offices (12), provided greater access to funding, information systems, and administrative support for CCM implementation (11,12,16,17).

## Discussion

The findings of these studies contribute to a qualitative understanding of the relationship between the application of CCM components and diabetes outcomes in US primary care settings. Although the original CCM has been critiqued for not adequately meeting the needs of diverse patient populations with diabetes (7), our systematic review supports the idea that CCM-based interventions are generally effective for managing diabetes in US primary care settings.

One meta-analysis (27) determined that no single component of the CCM was imperative for improved outcomes. However, it is important to determine the combination of components that will likely produce optimal patient and provider outcomes. Our review suggested that incorporating multiple components together in the same intervention can help facilitate better CCM implementation (eg, using the decision-support component to train providers on guidelines such as the ADA Standards of Care and using the delivery system design component to remodel the care delivery process to provide self-management support through DSME in PCP offices).

In several studies, organizational leaders in health care systems initiated system-level reorganizations that facilitated more comprehensive and coordinated diabetes care. Changing staff roles and responsibilities to more efficiently treat diabetes was 1 strategy that produced clinical benefits. Reorganized care can also support better training programs for patients to help them self-manage diabetes. Future system-level CCM reorganizations should create clear access points for providers to intervene with patients who are at risk for diabetes complications. Some organizations have already begun to do so. For example, the Rockwood Clinic Foundation revised its mission statement to include fundraising for research and development in new methods of chronic care delivery, which has resulted in increased funding for training materials, glucometers, blood pressure monitors, and laboratories (20).

In several studies (10–12,14,16–18), providing administrative support to train PCPs in implementing evidence-based care was associated with improved patient engagement that led to positive health outcomes. Future studies should examine the effects of continuing education for ADA Standards of Care and ICSI clinical guidelines on CCM decision support among providers. It is important to determine whether provider training delivered through telecommunication and distance learning technologies can provide ample decision-support training to PCPs. Another area worth investigating is whether the longitudinal use of decision support in different primary care practice settings (eg, private practices, community health centers, hospitals) improves patient outcomes.

Delivery system design was identified as an important strategy for integrating DSME into primary care settings through addressing patient barriers to care such as accessibility to DSME and availability of staff to assist with diabetes care (10). Our review supports the idea that DSME improves psychosocial and clinical outcomes. DSME fostered learning about proactive diabetes self-care practices and self-management skills. When ADA-accredited DSME occurs in primary care settings, PCPs are able to provide patients with personalized access to CDEs, who are likely funded through third-party health insurers (12). Offering DSME in primary care settings, rather than solely hospital settings, enhances the reach of such programs in a more intimate, socially supportive venue. Future DSME for primary care patients should continue to cover the ADA content areas (28) for diabetes self-management, and strategies for delivering DSME should be evaluated by assessing the comparative effectiveness of group- and individual-level DSME approaches.

Only 1 study in our review (24) conducted weekly, skill-based learning sessions for racial/ethnic minority groups on healthful cooking modifications for traditional foods and snacks. This type of culturally appropriate self-management support was associated with a greater number of participants who had an HbA1c measurement of less than 7% and a fewer number of participants who had an HbA1c measurement of greater than 10% (24). Other culturally tailored non-CCM interventions (29) have demonstrated larger absolute reductions in HbA1c than nontailored interventions. Given the large number of racial/ethnic minority populations in the United States who are at high risk for type 2 diabetes (eg, African Americans, Hispanics, American Indians, Asian Americans, Pacific Islanders) (30), future research should focus on culturally tailored DSME in primary care settings. Cultural factors (eg, food preparation, views of illness) should be considered when designing, implementing, and evaluating DSME for these underserved groups (31). It is also noteworthy that none of the reviewed studies addressed the needs of pediatric patients diagnosed with either type 1 or type 2 diabetes. Diabetes is becoming more common in children and adolescents (32); Rapley and Davidson (33) have advocated for the adoption of CCM programs aimed at adolescent patients with diabetes to help bridge the gap between pediatric and adult care.

More personalized, patient-centered interactions (eg. individual office visits) help patients and providers set behavioral and clinical goals that can be monitored through clinical information systems. Many studies (9,10,12,14,18,20, 21,23,24) used disease registries and electronic medical records to establish patient goals, monitor patient progress, and determine lapses in patient care. Assimilating clinical information systems into user-friendly, portable digital technologies (ie, smartphones, iPads) may enable patients and providers to view and respond to laboratory results more regularly. For older populations of chronic disease patients (the age group sampled in most of the reviewed studies), training programs on the use of digital technologies for diabetes self-management may reduce the anxiety and barriers to access that may currently exist (23,34). Involving patients in exploratory focus groups to inform the development of assistive technologies can customize educational technology and address usability concerns among unique patient populations (35). Future studies on diabetes self-management support within the broader CCM framework should attempt to refine the use of information and communications technologies to empower, engage, and educate patients (36).

Finally, community-level partnerships pooled human and fiscal resources to provide diabetes management services (11,12,16–18,20,24). However, strategies for using community resources and developing policies were described in only 7 studies. A meta-analysis (27) also found that few studies addressed the community resources and policies component of CCM. More public-private partnerships need to be developed between providers and community organizations to address barriers to care and explore culturally appropriate community-based services (eg, cooking classes, exercise programs, nutrition counseling, self-monitoring assistance) for underserved populations and neighborhoods. Other models have sought to improve the community resources and policies component of the CCM. The Innovative Care for Chronic Conditions (ICCC) model espoused by the World Health Organization (33,37) is comparable to the Expanded Chronic Care Model proposed by Barr and colleagues (7); it introduces prevention efforts, social determinants of health, and enhanced community participation as core components of chronic disease care. The ICCC has a larger focus on supporting “positive policy environments” (ie, partnerships, legislative frameworks, human resource allocation, leadership, and financing) in community and health care organizations (33,37). Future studies should investigate how different derivations of CCM components contribute to changes in diabetes care within primary care settings.

This study had several limitations. We used only a few search terms, so all relevant studies may not have been identified. Only 1 person selected the studies for inclusion in our review. Future studies should use the multiple-rater approach for study selection and data extraction as outlined by the Centre for Reviews and Dissemination systematic review guidelines (8). We did not conduct a meta-analysis because we did not have access to primary data, and the variability in study design did not allow us to pool data. Future research could include a meta-analysis of data (27) from randomized controlled trials to evaluate the methodological quality of quantitative studies that have tested the effectiveness of CCM for managing diabetes. 

In conclusion, our study provides evidence that CCM is effective in improving the health of people who have diabetes and receive care in primary care settings. The model accounts for health services at various levels in the diabetes care process. Positive clinical outcomes have been cited as indicators of CCM’s success in diabetes management (9–24). Far less emphasis has been placed on measuring the process outcomes of CCM that help lead to functional and clinical improvements. Process outcomes (eg, self-efficacy for disease management and clinical decision making, perceived social support, knowledge of diabetes self-care practices) are all indicators that need to be assessed. These assessments could enable health care administrators and professionals to determine how CCM could become further integrated into primary health care initiatives in diabetes.

## References

[1] Centers for Disease Control and Prevention. National diabetes fact sheet: national estimates and general information on diabetes and prediabetes in the United States, 2011. http://www.cdc.gov/diabetes/pubs/pdf/ndfs_2011.pdf. Accessed June 12, 2012.

[2] Centers for Disease Control and Prevention. National diabetes fact sheet: general information and national estimates on diabetes in the United States, 2007. http://www.cdc.gov/diabetes/pubs/pdf/ndfs_2007.pdf. Accessed June 12, 2012.

[3] Centers for Disease Control and Prevention. Chronic diseases and health promotion. National Center for Chronic Disease Prevention and Health Promotion; 2010. http://www.cdc.gov/chronicdisease/overview/index.htm#ref1. Accessed June 12, 2012.

[4] Wagner EH , Austin BT , Davis C , Hindmarsh M , Schaefer J , Bonomi A . Improving chronic illness care: translating evidence into action. Health Aff (Millwood) 2001;20(6):64–78. 10.1377/hlthaff.20.6.64 11816692

[5] Wagner EH , Davis C , Schaefer J , Von Korff M , Austin B . A survey of leading chronic disease management programs: are they consistent with the literature? Manag Care Q 1999;7(3):56–66. 10620960

[6] Bodenheimer T , Wagner EH , Grumbach K . Improving primary care for patients with chronic illness. JAMA 2002;288(14):1775–9. 10.1001/jama.288.14.1775 12365965

[7] Barr VJ , Robinson S , Marin-Link B , Underhill L , Dotts A , Ravensdale D , The expanded chronic care model: an integration of concepts and strategies from population health promotion and the chronic care model. Hosp Q 2003;7(1):73–82. 14674182 10.12927/hcq.2003.16763

[8] Centre for Reviews and Dissemination. Systematic reviews: CRD’s guidance for undertaking reviews in health care. Layerthorpe, York (UK): York Publishing Services Ltd; 2009.

[9] MacLean CD , Littenburg B , Gagnon M , Reardon M , Turner PD , Jordan C . The Vermont Diabetes Information System (VDIS): study design and subject recruitment for a cluster randomized trial of a decision support system in a regional sample of primary care practices. Clin Trials 2004;1(6):532–44. 10.1191/1740774504cn051oa 16279294 PMC2518939

[10] Siminerio LM , Piatt G , Zgibor JC . Implementing the chronic care model for improvements in diabetes care and education in a rural primary care practice. Diabetes Educ 2005;31(2):225–34. 10.1177/0145721705275325 15797851

[11] Piatt GA , Orchard TJ , Emerson S , Simmons D , Songer TJ , Brooks MM , Translating the chronic care model into the community: results from a randomized controlled trial of a multifaceted diabetes care intervention. Diabetes Care 2006;29(4):811–7. 10.2337/diacare.29.04.06.dc05-1785 16567820

[12] Siminerio LM , Piatt GA , Emerson S , Ruppert K , Saul M , Solano F , Deploying the chronic care model to implement and sustain diabetes self-management training programs. Diabetes Educ 2006;32(2):253–60. 10.1177/0145721706287156 16554429

[13] Smith SA , Shah ND , Bryant SC , Christianson TJ , Bjornsen SS , Giesler PD , Chronic care model and shared care in diabetes: randomized trial of an electronic decision support system. Mayo Clin Proc 2008;83(7):747–57. 10.4065/83.7.747 18613991

[14] Stuckey HL , Dellasega C , Graber NJ , Mauger DT , Lendel I , Gabbay RA . Diabetes nurse case manager and motivational interviewing for change (DYNAMIC): Study design and baseline characteristics in the chronic care model for type 2 diabetes. Contemp Clin Trials 2009;30(4):366–74. 10.1016/j.cct.2009.03.002 19328244 PMC2740652

[15] Schillinger D , Handley M , Wang F , Hammer H . Effects of self-management support on structure, process, and outcomes among vulnerable patients with diabetes: a three-arm practical clinical trial. Diabetes Care 2009;32(4):559–66. 10.2337/dc08-0787 19131469 PMC2660485

[16] Piatt GA , Anderson RM , Brooks MM , Songer T , Siminerio LM , Korytkowski MM , 3-year follow-up of clinical and behavioral improvements following a multifaceted diabetes care intervention: results of a randomized controlled trial. Diabetes Educ 2010;36(2):301–9. 10.1177/0145721710361388 20200284

[17] Piatt GA , Songer TJ , Brooks MM , Anderson RM , Simmons D , Orchard TJ , Impact of patient level factors on the improvement of the ABCs of diabetes. Patient Educ Couns 2011;82(2):266–70. 10.1016/j.pec.2010.04.005 20434290

[18] Stroebel RJ , Gloor B , Freytag S , Riegert-Johnson D , Smith SA , Huschka T , Adapting the chronic care model to treat chronic illness at a free medical clinic. J Health Care Poor Underserved 2005;16(2):286–96. 10.1353/hpu.2005.0041 15937392

[19] Khan MA , Evans AT , Shah S . Caring for uninsured patients with diabetes: designing and evaluating a novel chronic care model for diabetes care. J Eval Clin Pract 2010;16(4):700–6. 10.1111/j.1365-2753.2009.01178.x 20545806

[20] Benedetti R , Flock B , Pedersen S , Ahern M . Improved clinical outcomes for fee-for-service physician practices participating in a diabetes care collaborative. Jt Comm J Qual Saf 2004;30(4):187–94. 15085784 10.1016/s1549-3741(04)30020-1

[21] Coca A , Francis MD . Implementing the chronic care model in an academic setting: a resident’s perspective. Sem Med Pract 2007;10(1):1–8.

[22] Caruso LB , Clough-Gorr KM , Silliman RA . Improving quality of care for urban older people with diabetes mellitus and cardiovascular disease. J Am Geriatr Soc 2007;55(10):1656–62. 10.1111/j.1532-5415.2007.01320.x 17714460

[23] Lyles CR , Harris LT , Le T , Flowers J , Tufano J , Britt D , Qualitative evaluation of a mobile phone and web-based collaborative care intervention for patients with type 2 diabetes. Diabetes Technol Ther 2011;13(5):563–9. 10.1089/dia.2010.0200 21406018

[24] Liebman J , Heffernan D , Sarvela P . Establishing diabetes self-management in a community health center serving low-income Latinos. Diabetes Educ 2007;33(Suppl 6):132S–8S. 10.1177/0145721707304075 17620392

[25] American Diabetes Association. Standards of medical care in diabetes. Diabetes Care 2012;35(Suppl 1):S11–63. 10.2337/dc12-s011 22187469 PMC3632172

[26] Simmons D , Weblemoe T , Voyle J , Prichard A , Leakehe L , Gatland B . Personal barriers to diabetes care: lessons from a multi-ethnic community in New Zealand. Diabet Med 1998;15(11):958–64. 10.1002/(SICI)1096-9136(1998110)15:11<958::AID-DIA687>3.0.CO;2-9 9827851

[27] Tsai AC , Morton SC , Mangione CM , Keeler EB . A meta-analysis of interventions to improve care for chronic illnesses. Am J Manag Care 2005;11(8):478–88. 16095434 PMC3244301

[28] Funnell MM , Brown TL , Childs BP , Haas LB , Hosey GM , Jensen B , National standards for self-management education. Diabetes Care 2009;32(Suppl 1):S87–94. 10.2337/dc09-S087 19118294 PMC2613581

[29] Peek ME , Cargill A , Huang ES . Diabetes health disparities: a systematic review of health care interventions. Med Care Res Rev 2007;64(5 Suppl):101S–56S. 10.1177/1077558707305409 17881626 PMC2367214

[30] Centers for Disease Control and Prevention. Groups especially affected. Diabetes Public Health Resource; 2012. http://www.cdc.gov/diabetes/consumer/groups.htm. Accessed October 7, 2012.

[31] Stellefson ML , Hanik BW , Chaney BH , Chaney JD . Challenges for tailored messaging in health education. Am J Health Educ 2008;39(5):303–11.

[32] Centers for Disease Control and Prevention. Children and diabetes — more information. Diabetes Public Health Resource; 2012. http://www.cdc.gov/diabetes/projects/cda2.htm. Accessed October 7, 2012.

[33] Rapley P , Davidson PM . Enough of the problem: a review of time for health care transition solutions for young adults with a chronic illness. J Clin Nurs 2010;19(3-4):313–23. 10.1111/j.1365-2702.2009.03027.x 20500270

[34] Stellefson M , Chaney B , Chaney D . The digital divide in health education: myth or reality? Am J Health Educ 2008;39(2):106–12.

[35] Stellefson M , Chaney BH , Chaney JD . Using exploratory focus groups to inform the development of targeted COPD self-management education DVDs for rural patients. Int J Telemed Appl 2010;2010:450418. 20672021 10.1155/2010/450418 PMC2909711

[36] Hall AK , Stellefson M , Bernhardt JM . Healthy Aging 2.0: the potential of new media and technology. Prev Chronic Dis 2012;9:E67. 22405474 PMC3368698

[37] Epping-Jordan J , editor. Innovative care for chronic conditions: building blocks for action: global report (document no. WHO/NMC/CCH/0201). World Health Organization; 2002.

